# Characterization of Clinical Outcomes for Patients with Relapsed High-Risk Neuroblastoma After Autologous Stem Cell Transplant and External Beam Radiotherapy

**DOI:** 10.3390/cancers18030520

**Published:** 2026-02-05

**Authors:** Mathew Lin, Jie Jane Chen, Rochelle Bagatell, Sherif G. Shaaban, Benjamin J. Lerman, Suzanne Shusterman, Myrsini Ioakeim-Ioannidou, Torunn I. Yock, Paul J. Catalano, Hesham Elhalawani, Kathryn E. Dusenbery, Kieuhoa T. Vo, Mary S. Huang, Alison M. Friedmann, Lisa R. Diller, Karen J. Marcus, Shannon M. MacDonald, Stephanie A. Terezakis, Steve E. Braunstein, Christine E. Hill-Kayser, Daphne A. Haas-Kogan, Steven G. DuBois, Kevin X. Liu

**Affiliations:** 1 Department of Radiation Oncology, Brigham and Women’s Hospital, Dana-Farber Cancer Institute, Boston Children’s Hospital, Harvard Medical School, Boston, MA 02215, USAhelhalawani@bwh.harvard.edu (H.E.); kmarcus@mgb.org (K.J.M.);; 2 Department of Radiation Oncology, University of California at San Francisco and UCSF Benioff Children’s Hospital, San Francisco, CA 94143, USA; jiejanechen@mdanderson.org (J.J.C.);; 3 Department of Pediatrics, Division of Oncology, Children’s Hospital of Philadelphia and the Perelman School of Medicine, University of Pennsylvania, Philadelphia, PA 19146, USA; bagatellr@chop.edu; 4 Department of Radiation Oncology, University of Minnesota, Minneapolis, MN 55455, USA; sherifgamal1082@gmail.com (S.G.S.);; 5 Department of Pediatrics, UCSF Benioff Children’s Hospital and UCSF School of Medicine, San Francisco, CA 94143, USA; 6 Department of Pediatrics, Dana-Farber/Boston Children’s Cancer and Blood Disorders Center, Harvard Medical School, Boston, MA 02215, USA; 7 Department of Radiation Oncology, Massachusetts General Hospital, Harvard Medical School, Boston, MA 02114, USA; 8 Department of Data Science, Dana-Farber Cancer Institute, Boston, MA 02215, USA; 9 Department of Pediatric Oncology, Massachusetts General Hospital, Boston, MA 02114, USAafriedmann@mgh.harvard.edu (A.M.F.); 10 Department of Radiation Oncology, University of Pennsylvania and the Children’s Hospital of Philadelphia, Philadelphia, PA 19104, USA

**Keywords:** photon radiotherapy, proton radiotherapy, surgery, palliative radiotherapy, time to relapse

## Abstract

Background: Limited data inform the outcomes of patients with high-risk neuroblastoma (HR-NBL) who relapse after high-dose chemotherapy, autologous stem cell transplantation (ASCT), and external beam radiotherapy (EBRT). Methods: This is a multi-institutional retrospective study of 84 patients with HR-NBL diagnosed between 1997–2021 with a first recurrence after definitive upfront treatment, including ≥1 ASCT and EBRT. Site(s) of first relapse were defined with relation to a patient’s primary tumor location. Progression-free survival (PFS) and overall survival (OS) outcomes were analyzed using Kaplan–Meier curves and log-rank tests. Cox proportional hazard models were used for univariate and multivariable analyses. Results: Twenty-four patients had local recurrences with or without distant relapses (LR) and 60 had distant relapses only. The LR cohort had higher rates of *MYCN* amplification (70% vs. 36%, *p* = 0.016). At relapse, the LR cohort had lower rates of additional radiotherapy (32% vs. 61%, *p* = 0.029) and higher rates of additional surgery (29% vs. 5%, *p* = 0.005), with similar rates of chemotherapy for both cohorts. With a median follow-up after first relapse of 1.53 years (range: 0.03–15.82), there were no significant differences in interval PFS and OS between the cohorts. After controlling for age at diagnosis and pattern of recurrence, time to interval relapse ≥ 2 years was a significant predictor of improved OS (HR: 0.50, 95% CI: 0.29–0.85, *p* = 0.011). Conclusions: Patients with relapsed HR-NBL have poor outcomes with median OS < 2 years. Time to relapse was a significant predictor of OS.

## Simple Summary

Despite intensive multimodal treatment, patients with high-risk neuroblastoma (HR-NBL) remain at risk of relapse with an overall survival of approximately 50%. There is little data regarding outcomes for patients with relapsed HR-NBL, thus we sought to better characterize outcomes in a multi-institutional cohort of 84 patients with HR-NBL who relapsed after receiving the current standard of care, including autologous stem cell transplantation and external beam radiotherapy, stratified by patterns of first relapse. Patients with local recurrences with or without distant relapses had higher rates of *MYCN* amplification. For the entire cohort, overall survival remained poor after relapse with a median of <2 years with no difference when stratifying by patterns of first relapse. Multivariable analysis found that a time to interval relapse ≥ 2 years was a significant predictor of improved survival. Future studies are needed to validate these findings and identify additional prognostic clinical or molecular factors.

## 1. Introduction

Neuroblastoma is the most common extracranial solid tumor in childhood. Clinical, histologic, and molecular factors, such as age, histology, segmental chromosome aberrations (SCA), and *MYCN* amplification help risk-stratify patients and determine appropriate therapy [1]. Patients with high-risk neuroblastoma require multimodality treatment that includes chemotherapy, surgery, autologous stem cell transplant (ASCT), external beam radiation therapy (EBRT), and anti-GD2 immunotherapy [2,3,4,5,6,7]. Despite improvements in the treatment of patients with high-risk neuroblastoma, relapses remain common and 5-year overall survival (OS) is approximately 50% [2,5,7].

There are limited data regarding outcomes for patients with relapsed high-risk neuroblastoma after ASCT and EBRT—current standards of care [2,4,5]. Furthermore, few studies have explored whether the location of initial relapse influences outcomes [8]. Among studies examining the effect of site of relapse, Vo et al. reported a significantly lower 5-year OS rate among patients with combined local and distant relapse than among patients with isolated local recurrence or distant-only recurrence, after adjusting for age, stage, and *MYCN* amplification status [8]. Vo and colleagues were unable to assess detailed data regarding treatments received at diagnosis or relapse as registry data were mined for that study. Therefore, we conducted a study to investigate whether clinical outcomes are inferior for patients who had local recurrences after ASCT and radiation therapy compared to patients with distant-only recurrence, and to describe factors associated with OS.

## 2. Materials and Methods

The Institutional Review Boards at all institutions approved a multi-institutional retrospective study of 84 patients with high-risk neuroblastoma diagnosed between 1997–2021 who developed a recurrence after definitive upfront treatment for high-risk neuroblastoma. Patients were excluded if they did not receive at least one ASCT and EBRT to the primary site. Patients were also excluded if they had no follow-up after relapse. Medical records for all patients were reviewed. Data collection included: demographics at diagnosis and relapse, including age and sex; disease characteristics at diagnosis, including *MYCN* amplification status, histopathology classification and location of primary site; treatments after initial diagnosis, including chemotherapy, surgery, ASCT, and EBRT, and post-consolidation therapy. In addition, data at first relapse was also collected, including treatment after first relapse, including surgery, chemotherapy, and EBRT; and follow-up information, including date of last clinical follow-up, date of death, and date of second relapse. Induction chemotherapy was selected at the discretion of the treating physician as per or on study using well-established North American protocols [2,4,5,9,10,11,12]. Radiation dose and fields were also determined at the discretion of the treating physician as per or on study using the prior North American protocols [3,9,13]. Most patients received 21.6–22.8 Gy [9,13]; however, some patients received an additional 14.4 Gy boost for a total dose of 36 Gy on Children’s Oncology Group (COG) ANBL0532 or off study before results were reported [3,14].

Imaging was reviewed for all patients to determine the site(s) of first relapse. Locoregional recurrence was defined as recurrence at the primary site or within one nodal echelon beyond the disease present at diagnosis, as previously described [15]. All other sites of relapse were categorized as distant. Patients were classified as having local-only relapse, local and distant relapse, and distant-only relapse. Gross total resection was defined for patients with and without end-induction scans as per COG ANBL0532 (≤1 cm^3^ residual soft tissue density on end-induction scans) and COG A3973 (surgeon-defined > 90% resection), respectively [3,16].

For statistical analyses, STATA v15.0 (Stata Corp, College Station, TX, USA) and R v4.5.1. (R Core Team, Vienna, Austria) were used [17]. The primary objectives of this study were to define progression-free survival (PFS) and OS after first relapse, stratifying by location of relapse using Kaplan–Meier curves and log-rank tests. PFS was defined as the time from first relapse to second relapse or death. OS was defined as the time from first relapse to death. To identify factors associated with survival, Cox proportional hazard models were used for univariate analysis (UVA) and multivariable analysis (MVA). MVA analysis included variables that were significant for UVA as well as the location of relapse. Continuous variables were dichotomized at the median for each cohort except for age at diagnosis, which was dichotomized at 18 months given prior data [18]. For categorical variables, Fisher exact tests were performed, and the number of patients with unknown data is reported, but not included in the analysis. *p*-values < 0.05 were considered significant.

## 3. Results

### 3.1. Patient Characteristics

Of the 84 patients, 13 had local recurrence, 60 had isolated distant relapse, and 11 had combined local and distant relapses. Patients with local recurrence or combined local and distant relapses had similarities in clinical characteristics and outcomes. Therefore, these patients were grouped together as LR for subsequent analyses given the relatively small cohort sizes (Supplemental Tables S1 and S2, Supplemental Figures S1 and S2).

Baseline characteristics are presented in Table 1. The LR cohort had a median age at diagnosis of 3.31 years (range: 0.56–15.18) while the distant-relapse only cohort had a significantly older median age at diagnosis of 3.61 years (range: 0.62–19.73; *p* = 0.38). Median age at recurrence in the LR cohort was 5.14 years (range: 2.32–18.83) with a median time to relapse of 1.66 years (range: 0.61–4.38). In the distant-relapse only cohort, median age at recurrence was 6.48 years (range: 1.51–22.08) with a median time to relapse of 2.02 years (range: 0.71–10.05). *MYCN* amplification occurred more frequently in the LR cohort compared to the distant-relapse only cohort (70% vs. 36%, respectively; *p* = 0.016). No other baseline treatment characteristics differed significantly between the two groups. Of note, all patients < 18 months of age at diagnosis had tumor *MYCN* amplification (*p* < 0.001). In addition, age < 6 years at recurrence also was associated with *MYCN* amplification (64% vs. 27%, *p* = 0.002).

### 3.2. Treatment at First Relapse

Data regarding treatment at first relapse are presented in Table 2. The majority of patients received systemic therapy at first relapse, with similar rates between the two cohorts (*p* = 0.684). Many different systemic therapy regimens were used as first regimen at first relapse, but topotecan/cyclophosphamide or temozolomide/irinotecan with or without dinutuximab were the most common. While only a small number of patients underwent surgery at first relapse, significantly more patients in the LR cohort received surgery (29% vs. 5%, *p* = 0.005). At first relapse, significantly fewer patients in the LR cohort received radiotherapy to any lesion (primary tumor and/or metastatic site) (32% vs. 61%, *p* = 0.029). Only one patient received re-irradiation to a recurrent retroperitoneal mass.

### 3.3. Clinical Outcomes After First Relapse

**Figure 1:**
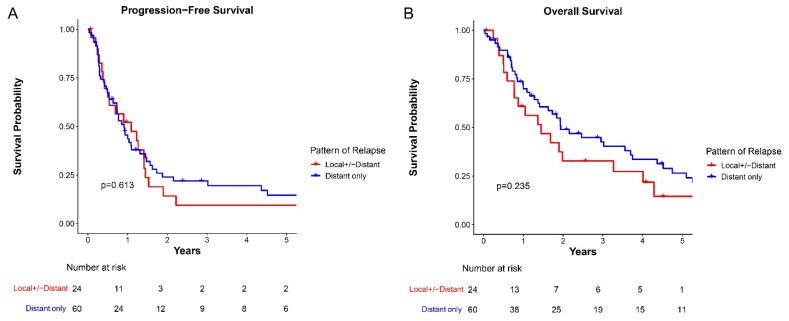
Survival outcomes after relapse stratified by patterns of relapse. (**A**) Progression-free survival stratified by patterns of relapse; (**B**) Overall survival stratified by patterns of relapse.

Median follow-up time after first relapse was 1.53 years (range: 0.03–15.82). There was no significant difference in PFS between the two cohorts (Figure 1A). Median OS in the LR cohort and distant-relapse only cohort were 1.45 years and 1.94 years, respectively (*p* = 0.230; Figure 1B). The 5-year estimated OS rates were 14.6 ± 8.5% for LR and 26.5 ± 6.4% for distant-only groups. Given that treatment options have evolved over time, especially the inclusion of post-consolidation anti-GD2 immunotherapy [19], we also investigated whether outcomes were different for patients diagnosed before 2010. We found that OS after first relapse was not significantly different for patients diagnosed between 1997–2009 and 2010–2021 (*p* = 0.875, Supplemental Figure S3). Nineteen patients received anti-GD2 immunotherapy at first relapse, but OS did not differ for these patients stratified by prior receipt of post-consolidation anti-GD2 immunotherapy (*p* = 0.657, Supplemental Figure S4).

We next sought to identify other factors associated with OS after first relapse using univariate and multivariable analyses. On univariate analyses, age ≥ 18 months at diagnosis (HR: 0.47, 95% CI: 0.24–0.93, *p* = 0.030), age ≥ 6 years at first recurrence (HR: 0.54, 95% CI: 0.33–0.89, *p* = 0.017), and time to relapse ≥ 2 years (HR: 0.46, 95% CI: 0.27–0.77, *p* = 0.004) were associated with improved OS (Table 3). No other characteristics during upfront or relapse treatment were predictive of OS, including chemotherapy, radiation therapy, or surgery undertaken at the time of recurrence.

Because age ≥ 18 months at diagnosis correlated with age ≥ 6 years at recurrence (*p* = 0.002), the two variables were separated in multivariable analyses. On multivariable analyses, time to relapse ≥ 2 years (HR: 0.50, 95% CI: 0.29–0.85, *p* = 0.011) remained a significant predictor of improved OS when controlling for age at diagnosis ≥ 18 months and pattern of recurrence (Table 4). There were similar findings when controlling for age ≥ 6 years at first recurrence and pattern of recurrence with a trend to significance for time to relapse (HR: 0.54, 95% CI: 0.30–1.00, *p* = 0.051, Table 4). Recurrence type (LR vs. distant-only) was not a significant predictor of OS in multivariable analyses.

**Figure 2:**
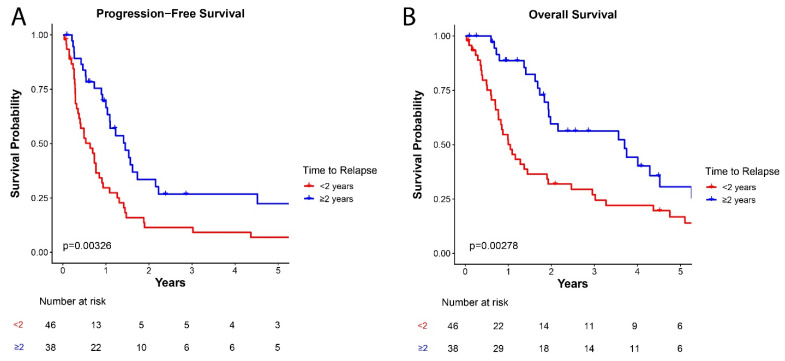
Survival outcomes after relapse stratified by time to relapse. (**A**) Progression-free survival stratified by time to relapse; (**B**) Overall survival stratified by time to relapse.

The two-year PFS for patients with time to relapse ≥ 2 years was 33.5 ± 8.4% compared to 11.4 ± 4.8% for those with time to relapse < 2 years (*p* = 0.003; Figure 2A). In addition, two-year OS for patients with time to relapse ≥ 2 years was 59.6 ± 8.7% compared to 31.9 ± 7.0% for those with time to relapse < 2 years (*p* = 0.003; Figure 2B).

## 4. Discussion

Few studies have examined outcomes for patients with relapsed high-risk neuroblastoma following a contemporary treatment paradigm including both ASCT and EBRT, particularly in the era of anti-GD2 chemoimmunotherapy for relapsed disease [20,21]. This is one of the largest studies to describe outcomes in patients with relapsed high-risk neuroblastoma after ASCT and EBRT with respect to outcomes after first relapse stratified by patterns of relapse. We found that patients with local or combined local and distant relapse had similar characteristics. There was no significant difference in PFS or OS between the LR cohort compared to the distant-only relapse cohort. On multivariable analyses, time to relapse ≥ 2 years was a significant predictor of improved OS when controlling for age at diagnosis and pattern of recurrence.

Few studies have specifically investigated outcomes at first relapse after intensive treatment for high-risk neuroblastoma. While Simon et al. found that intensive chemotherapy with second ASCT may benefit some patients, that study did not stratify by pattern of relapse [22]. Vo et al. used the International Neuroblastoma Risk Group (INRG) database to explore this question, including all patients with relapsed neuroblastoma. When specifically investigating outcomes for patients ≥ 18 months old with INSS stage 4 disease, those with combined local and distant failure had significantly worse 5-year OS compared to distant or local failure alone [8]. Median survival was numerically longer for patients with distant-only relapse, but the difference was not statistically significant. A prior study found that local recurrences correlated with worse survival [23]. Nonetheless, median survival was poor at < 2 years, similar to prior studies [24]. The PFS was also not different, likely due to similar rates of disease progression after first-line relapse therapy, which is primarily systemic therapy. We also found that fewer patients in the LR cohort received radiotherapy (32% vs. 61%; *p* = 0.029). This finding may reflect the challenges of re-irradiation, particularly within the abdomen. However, the relatively low dose of radiation used for neuroblastoma does leave sufficient organ tolerance to administer reirradiation without surpassing normal organ tolerance for most patients. Consideration of radioresistance may also influence providers’ decision-making, though prior studies demonstrate the efficacy of palliative radiotherapy for patients with recurrent and/or metastatic disease [25,26,27,28].

We found that upfront treatment characteristics did not predict OS; however, age at diagnosis ≥ 18 months, age at recurrence ≥ 6 years, and time to relapse ≥ 2 years were associated with improved OS on univariate analyses. Only time to relapse was a significant predictor in multivariable analyses. Time to relapse has been noted to be prognostic in other pediatric cancers [29,30,31]. Patients who have a longer time to relapse may have a more indolent disease and/or a disease more sensitive to treatment, resulting in more favorable responses to further treatment. Simon et al. found that patients who had longer remission time before first relapse had improved outcomes using cutoffs of 18 months and 24 months [22]. London et al. found that a shorter time to first relapse was significantly associated with poorer OS in a relapsed neuroblastoma cohort that included both high-risk and non-high risk patients [24], consistent with the association we observed in our high-risk cohort. Older age is known to correlate with worse outcomes for patients with neuroblastoma [24,32], thus it is surprising that age at diagnosis ≥ 18 months was associated with improved survival outcomes after relapse. This finding is likely driven by a correlation between *MCYN* amplification status and patients < 18 months at diagnosis. While prior studies included all patients with neuroblastoma [24,32], our study only includes patients with high-risk neuroblastoma. *MYCN* amplification is the most common factor that would classify a patient < 18 months at diagnosis as high-risk, and this is reflected in all 13 patients with age at diagnosis < 18 months in our cohort having tumors with *MYCN* amplification. There was a trend to worse survival for patients with tumors harboring *MYCN* amplification (*p* = 0.107) within our cohort. Other studies have found similar associations between *MYCN* amplification and inferior survival after relapse [33]. Thus, other disease or molecular factors, such as *MYCN* amplification, independent of age at diagnosis, may be driving this finding. Age at recurrence ≥ 6 years was a significant factor on univariate analysis, but not on multivariable analyses. The correlation of age at recurrence with outcomes is likely explained by other factors, as patients with older age at recurrence include patients who had a longer disease control period and fewer older patients who had *MYCN* amplified tumors. Our findings demonstrate that time to relapse continues to be a significant predictor of OS in patients with relapsed high-risk neuroblastoma after contemporary first-line therapy, including ASCT and EBRT.

Limitations of our study include those inherent in the retrospective design. While this is a large multi-institutional study for relapsed high-risk neuroblastoma, the size of our study remains limited, particularly for patients with local only or local and distant relapse. Future studies with larger sample sizes are needed to better understand whether patients with local versus combined local and distant recurrence are truly similar in terms of outcomes. In addition, patients were treated over many decades and there are likely changes in treatment over the different eras, including changes in relapse treatment options [34,35], that could influence the results and cannot be fully captured in our analysis. Of note, OS was not different in our analysis when stratifying by treatment era. Our cohort had incomplete molecular profiling because many patients were also treated before detailed molecular testing was routinely performed. Thus, we are not able to associate molecular findings, such as SCA [36], with outcomes in this study. There are other factors, such as performance status, tolerance to prior therapy, and goals of care, which can influence physician and family decision-making regarding treatment options at relapse, but cannot be fully captured in this study. Furthermore, there can be bias due to our institutions being referral centers, and not all patients received treatment after first relapse at our institutions. More granular data regarding therapy administered post-relapse were not uniformly available for further analysis. Future studies are still needed to further understand the optimal treatment regimens at time of relapse and examine whether other factors, such as molecular or genetic findings, may be associated with survival.

## 5. Conclusions

We report findings from one of the largest multi-institutional studies regarding outcomes for patients with relapsed, high-risk neuroblastoma with a focus on a modern cohort in which all patients received ASCT and EBRT as part of definitive upfront therapy prior to relapse. Our findings suggest that patients with relapsed high-risk neuroblastoma continue to have poor outcomes with median OS between 1–2 years. Pattern of first relapse was not prognostic for outcomes after relapse. Furthermore, time to relapse was a significant predictor of OS. Future prospective studies are needed to validate these findings and further characterize factors such as molecular profiles that correlate with outcomes.

## Figures and Tables

**Table 1:** Clinical Characteristics at Diagnosis Stratified by Patterns of Relapse.

Characteristic		Recurrence Type		*p*-Value
		Local and Local/Distant *n* = 24	Distant Only *n* = 60	
Sex				
	Male	17 (71%)	33 (55%)	0.223
	Female	7 (29%)	27 (45%)	
Age at diagnosis				
	<18 months	5 (21%)	8 (13%)	0.505
	≥18 months	19 (79%)	52 (87%)	
INSS stage				
	3	2 (8%)	2 (3%)	0.574
	4	22 (92%)	58 (97%)	
*MYCN* amplification				
	Yes	14 (70%)	19 (36%)	0.016 *
	No	6 (30%)	34 (64%)	
	Unknown	4	7	
Shimada histology				
	Favorable	0 (0%)	4 (7%)	0.567
	Unfavorable	19 (100%)	50 (93%)	
	Unknown	5	6	
Autologous stem cell transplant				
	Single	8 (33%)	32 (53%)	0.146
	Tandem	16 (67%)	28 (47%)	
Degree of resection				
	GTR	14 (58%)	41 (68%)	0.450
	STR	10 (42%)	19 (32%)	
Induction chemotherapy				
	ANBL0532	6 (25%)	31 (52%)	0.073
	34DAT	6 (25%)	12 (20%)	
	Other	12 (50%)	17 (28%)	
Primary site				
	Adrenal/abdominal	23 (96%)	54 (90%)	0.667
	Other	1 (4%)	6 (10%)	
I131 MIBG therapy				
	Yes	3 (12%)	13 (22%)	0.376
	No	21 (88%)	46 (78%)	
	Unknown	0	1	
Primary site RT technique				
	Photon	19 (79%)	38 (63%)	0.201
	Proton	5 (21%)	22 (37%)	
RT to metastatic sites				
	Yes	3 (12%)	18 (31%)	0.100
	No	21 (88%)	40 (69%)	
	Unknown	0	2	
Anti-GD2 immunotherapy				
	Yes	11 (58%)	37 (67%)	0.579
	No	8 (42%)	18 (33%)	
	Unknown	5	5	

Abbreviations: INSS, The International Neuroblastoma Staging System; MIBG, metaiodobenzylguanidine; RT, radiotherapy. * *p* < 0.05.

**Table 2:** Treatment Characteristics at First Relapse.

Characteristic		Recurrence Type		*p*-Value
		Local and Local/Distant *n* = 24	Distant Only *n* = 60	
Chemotherapy				
	Yes	21 (88%)	54 (90%)	0.710
	No	3 (12%)	6 (10%)	
Systemic therapy regimen				
	Topotecan	0 (0%)	2 (4%)	0.211
	Topotecan, cyclophosphamide	10 (48%)	11 (20%)	
	Temozolomide, irinotecan	4 (19%)	14 (26%)	
	Temozolomide, irinotecan, dinutuximab	4 (19%)	11 (20%)	
	Other	3 (14%)	16 (30%)	
Radiation to primary and/or metastatic site(s)				
	Yes	8 (32%)	36 (61%)	0.029 *
	No	16 (64%)	23 (39%)	
	Unknown	0	1	
Surgery				
	Yes	7 (29%)	3 (5%)	0.005 *
	No	17 (71%)	57 (95%)	

* *p* < 0.05.

**Table 3:** Univariate Analysis for Factors Associated with Overall Survival.

	Hazard Ratio (95% CI)	*p*-Value
Sex Male Female	Ref. 0.92 (0.55–1.53)	0.747
Age at Diagnosis <18 months ≥18 months	Ref. 0.47 (0.24–0.93)	0.030 *
INSS Stage 3 4	Ref. 0.65 (0.20–2.09)	0.471
*MYCN* amplification No Yes	Ref. 1.56 (0.91–2.67)	0.107
Shimada histology Favorable Unfavorable	Ref. 2.51 (0.61–10.38)	0.203
Primary site Other Abdominal/adrenal	Ref. 1.06 (0.42–2.67)	0.894
Induction chemotherapy at diagnosis 34-DAT ANBL0532 Other	Ref. 1.34 (0.71–2.52) 1.13 (0.57–2.23)	0.366 0.726
Extent of resection at diagnosis GTR STR	Ref. 0.69 (0.40–1.19)	0.178
I131 MIBG therapy during upfront therapy No Yes	Ref. 1.03 (0.52–2.04)	0.939
Autologous stem cell transplant Single Tandem	Ref. 1.03 (0.62–1.70)	0.908
Primary site radiation modality Photon Proton	Ref. 1.52 (0.86–2.67)	0.149
Consolidative radiation to metastatic sites No Yes	Ref. 1.20 (0.69–2.11)	0.517
Anti-GD2 immunotherapy No Yes	Ref. 0.78 (0.45–1.35)	0.369
Pattern of first relapse Local or local/distant Distant only	Ref. 0.71 (0.41–1.25)	0.237
Age at first recurrence <6 years ≥6 years	Ref. 0.54 (0.33–0.89)	0.017 *
Time to relapse <2 years ≥2 years	Ref. 0.46 (0.27–0.77)	0.004 *
Chemotherapy at first relapse No Yes	Ref. 0.52 (0.23–1.14)	0.103
Radiotherapy at first relapse No Yes	Ref. 0.78 (0.47–1.29)	0.337
Surgery at first relapse No Yes	Ref. 0.77 (0.37–1.62)	0.494

Abbreviations: INSS, The International Neuroblastoma Staging System; MIBG, metaiodobenzylguanidine; RT, radiotherapy. * *p* < 0.05.

**Table 4:** Multivariable Analyses for Factors Associated with Overall Survival.

	Model 1		Model 2	
Variable	Hazard Ratio (95% CI)	*p*-Value	Hazard Ratio (95% CI)	*p*-Value
Age at diagnosis <18 months ≥18 months	Ref. 0.66 (0.30–1.43)	0.291		
Age at first recurrence <6 years ≥6 years			Ref. 0.74 (0.41–1.34)	0.327
Time to relapse <2 years ≥2 years	Ref. 0.50 (0.29–0.85)	0.011 *	Ref. 0.54 (0.30–1.00)	0.051
Pattern of relapse Local or local/distant Distant only	Ref. 0.87 (0.85–1.61)	0.653	Ref. 0.76 (0.44–1.34)	0.347

* *p* < 0.05.

## Data Availability

The data are not publicly available due to privacy or ethical restrictions. The data that support the findings of this study are available on request from the corresponding author.

## Supplementary Materials

The following supporting information can be downloaded at: https://www.mdpi.com/article/10.3390/cancers18030520/s1, Table S1. Clinical characteristics at diagnosis stratified by local-only, local and distant, and distant-only relapse; Table S2: Treatment characteristics at relapse stratified by local-only, local and distant, and distant-only relapse; Figure S1. Progression-free survival stratified by local-only, local and distant, and distant-only relapse; Figure S2. Overall survival stratified by local-only, local and distant, and distant-only relapse; Figure S3. Overall survival stratified by year of diagnosis; Figure S4. Overall survival for patients receiving anti-GD2 immunotherapy at first relapse stratified by receipt of post-consolidation anti-GD2 immunotherapy.
