# Supplementary material for: Characterization of Clinical Outcomes for Patients with Relapsed High-Risk Neuroblastoma After Autologous Stem Cell Transplant and External Beam Radiotherapy
Source: Cancers (Basel). 2026 Feb 5;18(3):520. doi: 10.3390/cancers18030520 (PMC12897123; doi:10.3390/cancers18030520)
Supplement: Supplementary file 1 [file cancers-18-00520-s001.zip › cancers-4140922-supplementary.pdf]

Supplemental Table S1. Clinical characteristics at diagnosis stratified by local-only, local and distant, and distant-only relapse.

Characteristic	Recurrence Type		
	**Local Only n = 13**	**Distant Only n = 60**	**Local and Distant n = 11**
Age at diagnosis, median (range) in years	2.15 (0.80 - 5.96)	3.61 (0.63 - 19.73)	4.14 (0.57 - 15.18)
Age at recurrence, median (range) in years	3.19 (2.32 - 9.04)	6.48 (1.51 - 22.08)	5.72 (2.37 - 18.83)
Time to relapse, median (range) in years	1.51 (0.61 - 4.38)	2.02 (0.71 - 10.05)	1.74 (1.16 - 3.65)
Sex			
Male	8	33	9
Female	5	27	2
Age at diagnosis			
<18 months	3	8	2
≥18 months	10	52	9
INSS stage			
3	1	2	1
4	12	58	10
MYCN amplification			
Yes	7	19	7
No	4	34	2
Unknown	2	7	2
Shimada histology			
Favorable	0	4	0
Unfavorable	12	50	7
Unknown	1	6	4
Autologous stem cell transplant			
Single	5	32	3
Tandem	8	28	8
Degree of resection			
GTR	7	41	7
STR	6	19	4
Unknown	0	0	1
Induction chemotherapy			
ANBL0532	4	31	2
34DAT	3	12	3
Other	6	17	6
Primary site			
Adrenal/abdominal	12	54	11
Other	1	6	0
MIBG therapy			
Yes	2	13	1
No	11	46	10
Primary site RT technique			
Photon	12	38	7
Proton	1	22	4
RT to metastatic sites			
Yes	3	18	0
No	10	40	11
Unknown	0	2	0
Anti-GD2 immunotherapy			
Yes	6	37	5
No	4	18	4
Unknown	3	5	2

Abbreviations: INSS, The International Neuroblastoma Staging System; MIBG, metaiodobenzylguanidine; RT, radiotherapy.

Supplemental Table S2: Treatment characteristics at relapse stratified by local-only, local and distant, and distant-only relapse

Characteristic	Recurrence Type		
	**Local Only n = 13**	**Distant Only n = 60**	**Local and Distant n = 11**
Chemotherapy			
Yes	10	54	11
No	3	6	0
Chemotherapy regimen			
Topotecan only	0	2	0
Topotecan, cyclophosphamide	3	11	7
Temozolomide, irinotecan	3	14	1
Temozolomide, irinotecan, dinutuximab	2	11	2
Other	2	16	1
None	3	6	0
Radiation			
Yes	5	36	3
No	8	23	8
Unknown	0	1	0
Surgery			
Yes	6	3	1
No	7	57	10

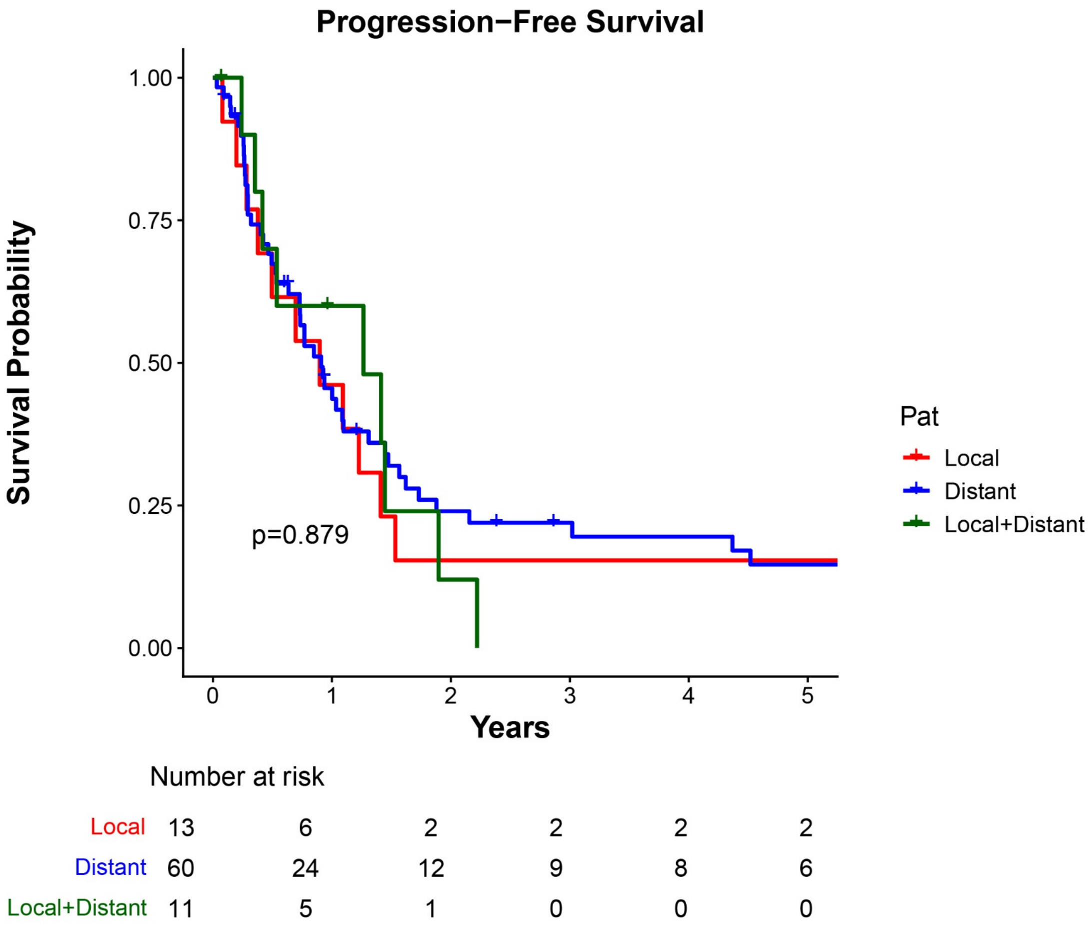

Supplemental Figure S1. Progression-free survival stratified by local-only, local and distant, and distant-only relapse.

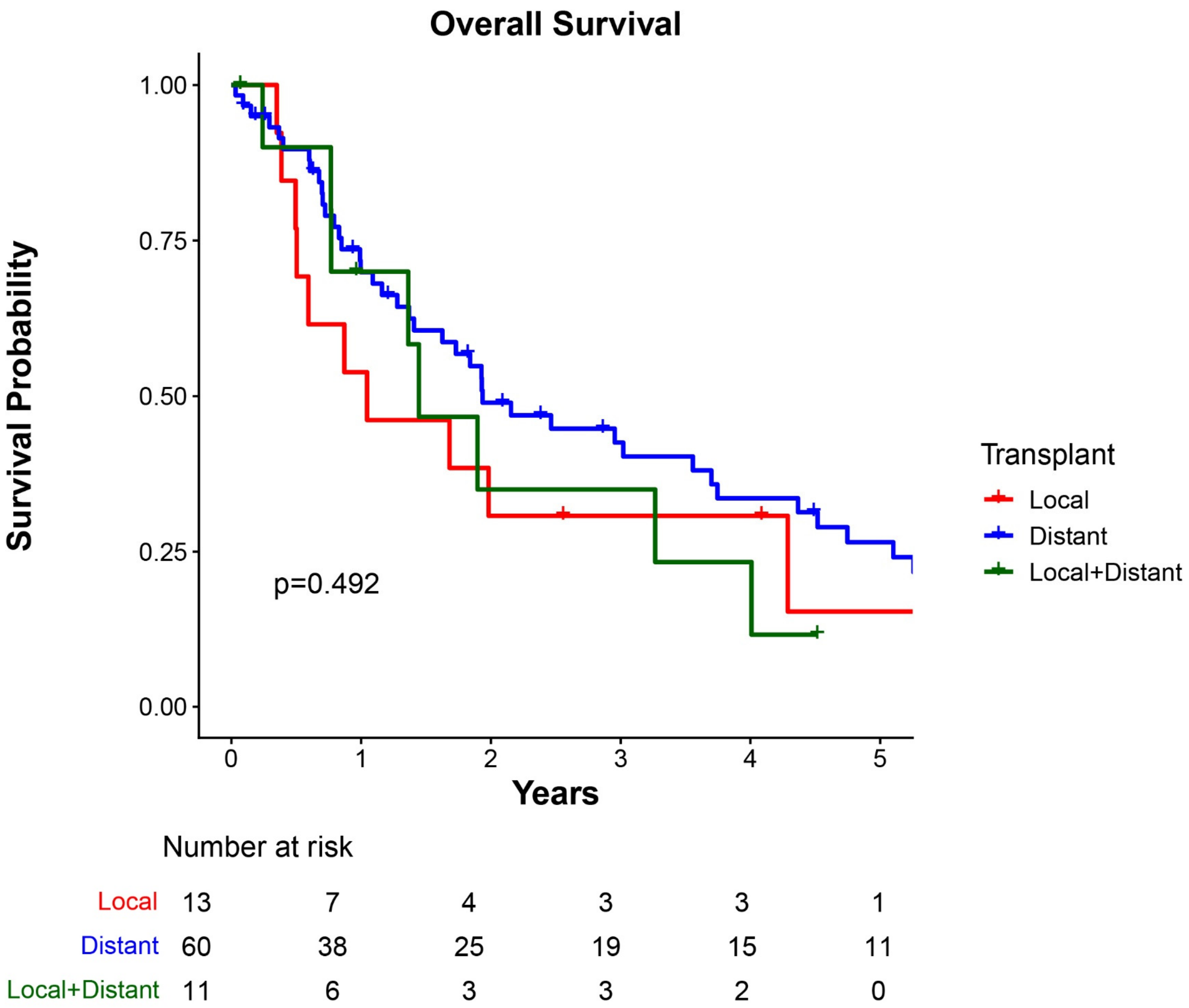

Supplemental Figure S2. Overall survival stratified by local-only, local and distant, and distant-only relapse.

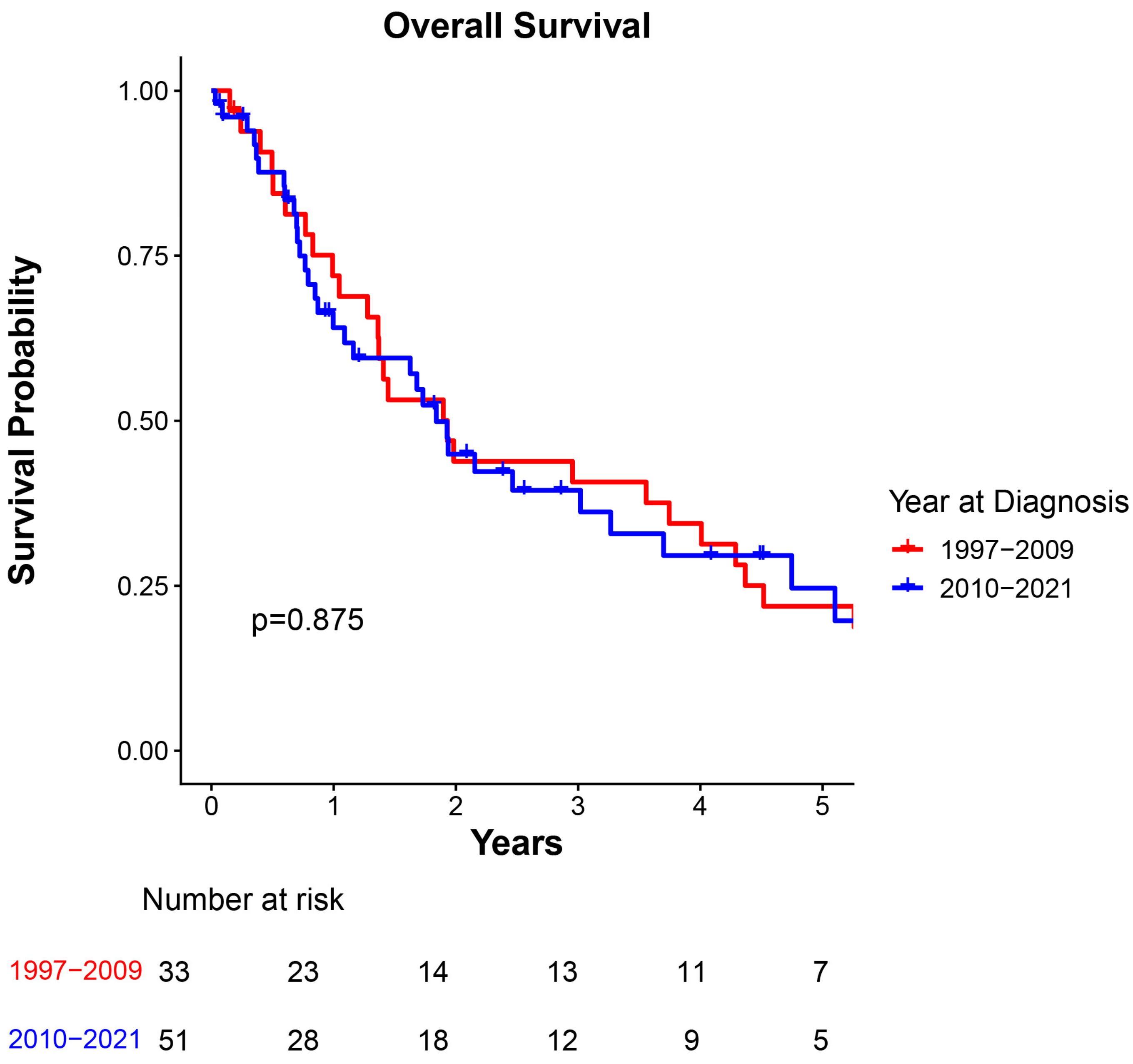

Supplemental Figure S3. Overall survival stratified by year of diagnosis.

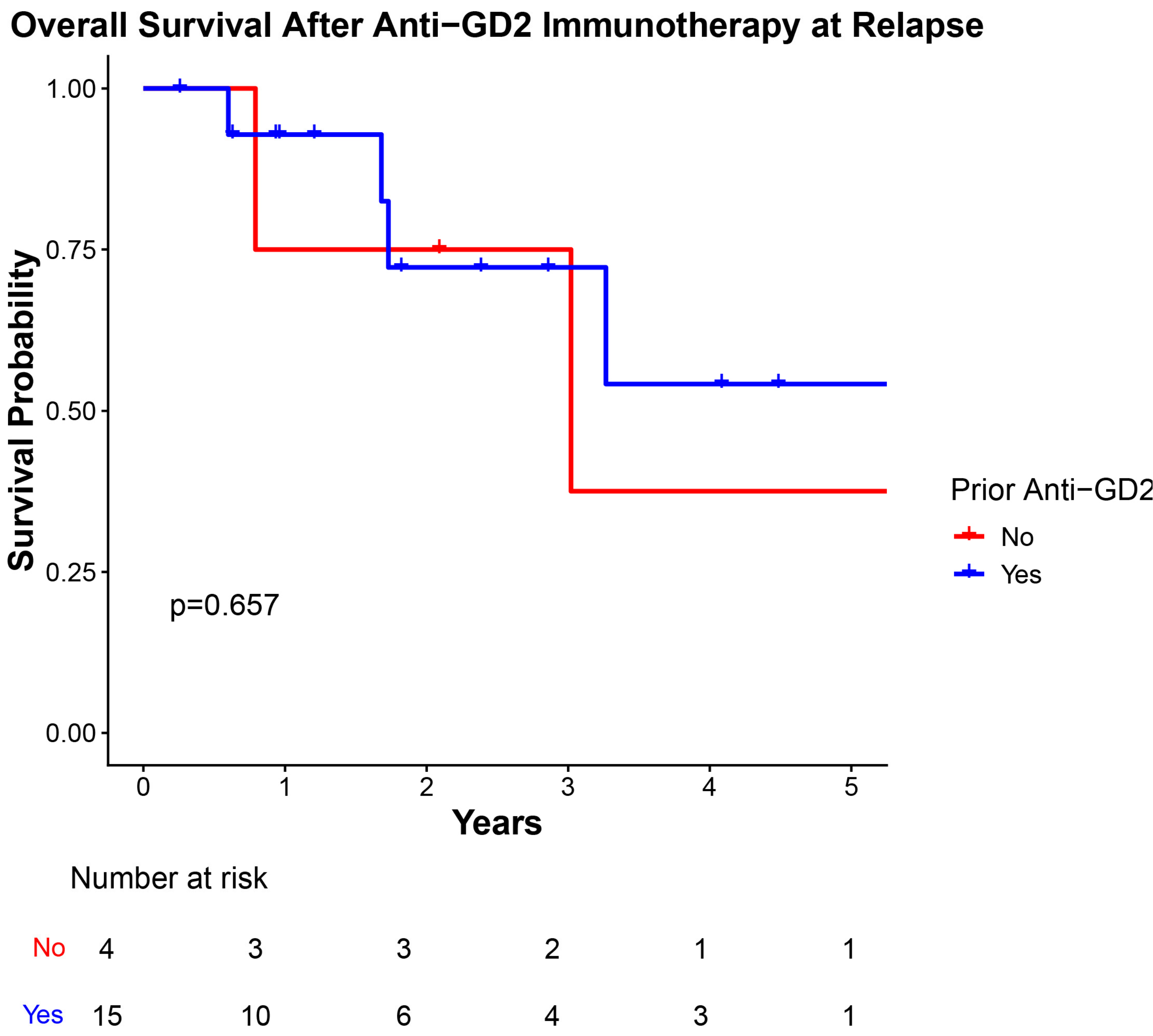

Supplemental Figure S4. Overall survival for patients receiving anti-GD2 immunotherapy at first relapse stratified by receipt of post-consolidation anti-GD2 immunotherapy.
